# Additive roles of antiferromagnetically coupled elements in the magnetic proximity effect in the GdFeCo/Pt system

**DOI:** 10.1038/s41598-024-60076-9

**Published:** 2024-04-25

**Authors:** Jung Yun Kee, Kook Tae Kim, In Hak Lee, Ilwan Seo, Jun-Young Chang, Ah-Yeon Lee, Woo-suk Noh, Young Jun Chang, Seung-Young Park, Sug-Bong Choe, Duck-Ho Kim, Kyoung-Whan Kim, Yongseong Choi, Dong Ryeol Lee, Jun Woo Choi

**Affiliations:** 1 Center for Spintronics, Korea Institute of Science and Technology (KIST), Seoul, 02792 Korea; 2 Department of Physics, Soongsil University, Seoul, 06978 Korea; 3 Department of Physics and Astronomy, Seoul National University, Seoul, 08826 Korea; 4 Center for Research Equipment, Division of Scientific Instrumentation & Management, Korea Basic Science Institute (KBSI), Daejeon, 34133 Korea; 5 Korea Foundation for Max Planck POSTECH/Korea Research Initiative, Pohang, 37673 Korea; 6 Department of Physics, University of Seoul, Seoul, 02504 Korea; 7 Center for Scientific Instrumentation, Division of Scientific Instrumentation & Management, Korea Basic Science Institute (KBSI), Daejeon, 34133 Korea; 8 Department of Physics, Yonsei University, Seoul, 03722 Korea; 9 Advanced Photon Source, Argonne National Laboratory, Argonne, IL 60439 USA

**Keywords:** Ferromagnetism, Magnetic properties and materials, Spintronics, Surfaces, interfaces and thin films, Magnetic properties and materials

## Abstract

Interfacial magnetic interactions between different elements are the origin of various spin-transport phenomena in multi-elemental magnetic systems. We investigate the coupling between the magnetic moments of the rare-earth, transition-metal, and heavy-metal elements across the interface in a GdFeCo/Pt thin film, an archetype system to investigate ferrimagnetic spintronics. The Pt magnetic moments induced by the antiferromagnetically aligned FeCo and Gd moments are measured using element-resolved x-ray measurements. It is revealed that the proximity-induced Pt magnetic moments are always aligned parallel to the FeCo magnetic moments, even below the ferrimagnetic compensation temperature where FeCo has a smaller moment than Gd. This is understood by a theoretical model showing distinct effects of the rare-earth Gd 4*f* and transition-metal FeCo 3*d* magnetic moments on the Pt electronic states. In particular, the Gd and FeCo work in-phase to align the Pt moment in the same direction, despite their antiferromagnetic configuration. The unexpected additive roles of the two antiferromagnetically coupled elements exemplify the importance of detailed interactions among the constituent elements in understanding magnetic and spintronic properties of thin film systems.

## Introduction

Considerable efforts have been made to utilize ferrimagnetic materials in spintronics^1–6^, owing to speed and density advantages that ferrimagnet-based spin devices offer over conventional ferromagnet-based systems. A well-known family of ferrimagnets is rare-earth (RE) transition-metal (TM) alloys (e.g., GdFe, TbCo). In RE–TM ferrimagnets, the RE magnetic moments mainly originate from the localized 4*f* electrons with a small contribution from the 5*d* electrons, whereas the TM exhibits itinerant ferromagnetism originating from the 3*d* electrons; the magnetic moments of the TM elements and RE elements are antiferromagnetically coupled^7^. The net magnetization of a RE–TM ferrimagnet is highly temperature-dependent as the magnetic moments of the RE and TM elements have different temperature dependence. Typically, the RE magnetic moment is larger (smaller) than the TM magnetic moment below (above) the ferrimagnetic compensation temperature (*T*_M_), such that the dominant magnetic element is interchanged across *T*_M_^1,8^.

**Figure 1:**
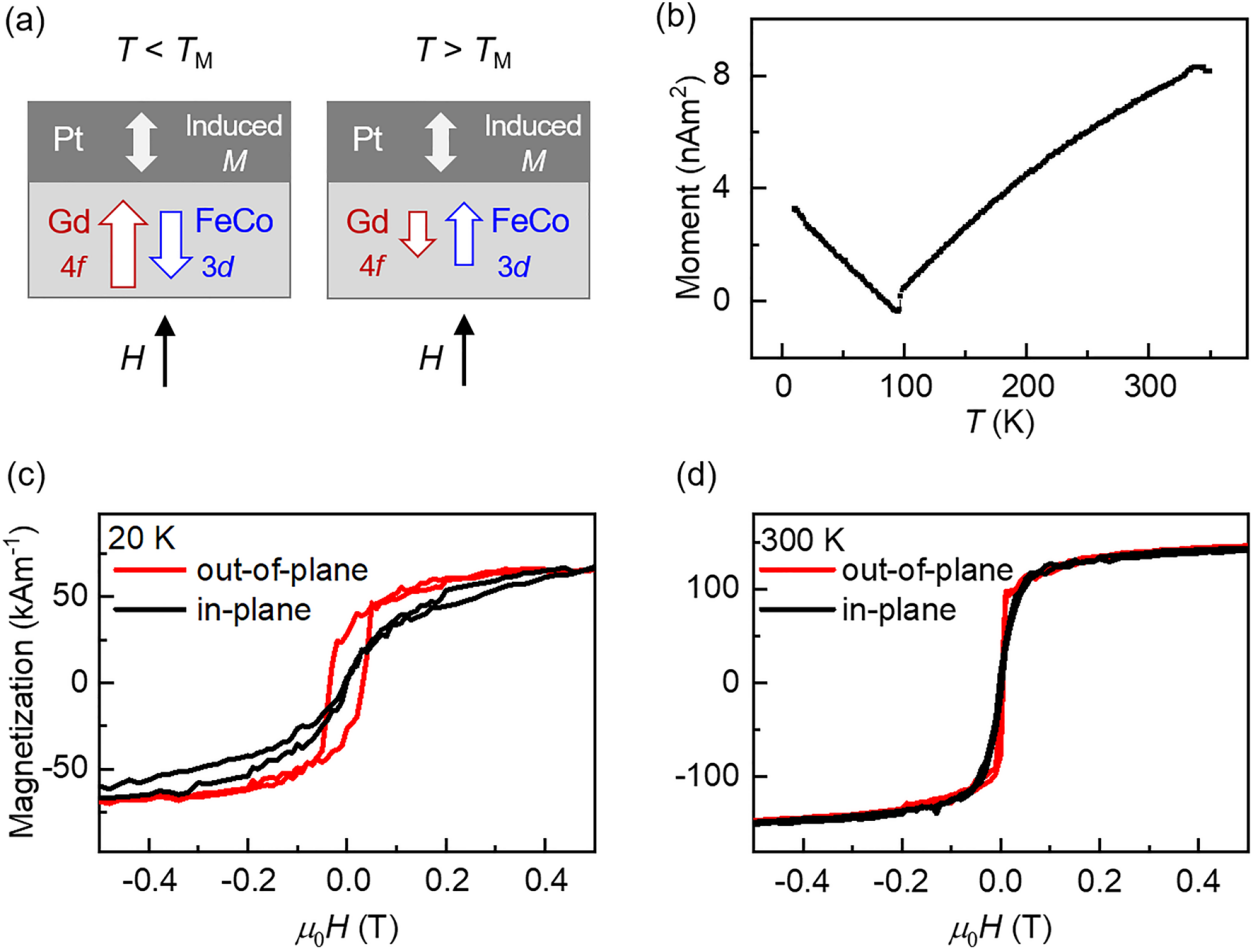
Elemental magnetic coupling and magnetic properties of the GdFeCo/Pt film. (**a**) Schematic diagram of the magnetic moment alignment in GdFeCo/Pt. (**b**) Temperature-dependent magnetization curve of GdFeCo/Pt, measured with 0.2 T out-of-plane field. Disappearance of the moment at approximately 100 K indicates the ferrimagnetic *T*_M_. (**c, d**) Magnetic hysteresis loops of GdFeCo/Pt, measured with the magnetic field applied out-of-plane (red) and in-plane (black). Measurements done at (**c**) *T* = 20 K and (**d**)* T* = 300 K. The magnetization in (**c**) and (**d**) is normalized by the volume of the film.

In bilayer systems consisting of a RE–TM ferrimagnet and a heavy-metal (HM), many novel spintronics phenomena originating from the RE–TM/HM interface emerge. The antiferromagnetic coupling between the RE and TM atoms along with the presence of large spin–orbit coupling in the HM result in intriguing spin-transport properties, such as fast domain wall dynamics^9,10^ and vanishing skyrmion Hall effect^11,12^. In certain RE–TM/HM systems, the interfacial electronic hybridization and exchange interaction can induce a magnetic moment in the normally non-magnetic HM, i.e., the so-called magnetic proximity effect (MPE)^13–21^. In the presence of MPE, the initially spin-degenerate energy bands in the HM can become spin-split. In this case, the various spin-split energy bands in the system can lead to many competing spin-dependent interactions between the electron orbitals with large DOS (e.g., 3*d* TM, 4*f* RE, 5*d* HM), which can result in the modulation of the magnetic and spin-transport properties of the system^17–21^. Nevertheless, the MPE induced by multi-elemental materials (e.g., RE–TM ferrimagnets) is not well-understood. The difficulty lies in the existence of multiple magnetic elements contributing to the MPE, and hence, it is unclear if the induced magnetic moment in the HM will couple strongly with a certain elemental (RE or TM) magnetic moment, simply align with the net magnetic moment (RE + TM), or be determined by another physical mechanism (see Fig. 1a for schematics). The understanding of MPE induced by a RE–TM ferrimagnet, particularly the distinct roles of the RE and TM moments, requires theoretical and experimental examinations of elemental magnetic coupling, which remains elusive so far.

In this study, we investigate the elemental magnetic coupling in a RE–TM/HM thin film system. Element-resolved magnetic moments in Gd_100−*x−y*_Fe_*x*_Co_*y*_/Pt films are measured using x-ray magnetic circular dichroism (XMCD) and x-ray resonant magnetic reflectivity (XRMR). Gd_100−*x−y*_Fe_*x*_Co_*y*_ is an exemplary RE–TM ferrimagnet, in which the ferromagnetically coupled Fe and Co magnetic moments are always anti-parallel aligned with the Gd magnetic moments. Gd_100−*x−y*_Fe_*x*_Co_*y*_ exhibits composition-dependent magnetic properties, and in particular, the compensation temperature *T*_M_ is highly dependent on the Gd content^8,22^. Below (above) the Gd_100−*x−y*_Fe_*x*_Co_*y*_* T*_M_, the Gd (FeCo) moment aligns parallel to the applied field. By performing x-ray measurements across the *T*_M_, we analyze the orientation of proximity-induced Pt magnetic moments relative to the Gd and FeCo moments. The Pt-specific magnetic data shows that the induced Pt moments are always aligned parallel to the FeCo magnetic moments, regardless of the FeCo having a smaller (below *T*_M_) or larger (above *T*_M_) magnetic moment than Gd.

The experimental observations are understood by a simple yet general principle which we call band repulsion. At the Gd_100−*x−y*_Fe_*x*_Co_*y*_–Pt interface, the Pt electronic states, originally spin-independent, are hybridized with the spin-dependent electronic states in the magnetic elements and become spin-split. We develop simple rules of the spin-dependent energy-splitting and conclude that the FeCo (Gd) elements induce magnetic moments in Pt which are parallel (anti-parallel) to their own magnetic moments. We first note that the Gd 4*f* electronic states are nearly half-filled in contrast to the FeCo 3*d* states whose spin polarization is mediated by the Gd 5*d* states. Our theoretical model shows that the half-filled nature of the Gd 4*f* electronic states actively contribute to the induced Pt moments being aligned *anti-parallel* to its own moments. In other words, both Gd and FeCo elements work in-phase to align the induced Pt magnetic moment in the same direction (parallel to the FeCo moments), despite the antiferromagnetic coupling between them. Our analyses shed light on the complex nature of the physics behind the MPE in multi-elemental heterostructures, which is far beyond simplistic speculations on the predominant role of a specific elemental moment or the net magnetic moment. In particular, the cooperative roles of the antiferromagnetically-coupled RE and TM elements imply that the proximity-induced HM moment can be decoupled from the net ferrimagnetic moment in certain RE–TM/HM systems. Furthermore, our results suggest that careful material/compositional selection of the RE–TM can lead to tunable magnetic interactions in RE–TM/HM systems, relevant to spin-transport phenomena in such systems.

## Results and discussion

### Magnetic characterization of GdFeCo/Pt

A SiN(5 nm)/Gd_23_Fe_67.4_Co_9.6_(8 nm)/Pt(5 nm) film sputter-deposited on a Si substrate with a thermally oxidized SiO_2_(100 nm) layer (GdFeCo/Pt hereafter; Pt is the top-most layer), is used for the study (see Methods for film growth process). The temperature-dependent magnetization curve of the GdFeCo/Pt film, measured by vibrating-sample-magnetometry with 0.2 T out-of-plane field applied, shows zero magnetization at approximately 100 K (Fig. 1b), confirming that the GdFeCo is ferrimagnetic with *T*_M_ being approximately 100 K^23,24^. Below (above) *T*_M_, the magnetic moment of Gd is stronger (weaker) than that of FeCo as depicted in Fig. 1a^25,26^, such that the net magnetization of the GdFeCo is non-zero. The out-of-plane magnetic hysteresis loops, measured at representative temperatures below and above *T*_M_ (Fig. 1c,d), suggest the existence of perpendicular magnetic anisotropy (PMA) in GdFeCo/Pt.

### Element resolved magnetic moments of GdFeCo/Pt

In order to investigate the proximity-induced magnetism of Pt and the magnetic coupling between the GdFeCo and Pt, elemental magnetic properties are characterized by performing XMCD at the Gd, Fe, and Pt x-ray absorption edges (see Methods). The XMCD spectra, defined by the difference between the x-ray absorption spectra measured with x-ray helicity parallel and anti-parallel to the applied magnetic field, provides a measure of the element-specific magnetic moment aligned with the magnetic field direction. Out-of-plane and in-plane XMCD measurements are performed at *T* = 10 or 20 K and 300 K, which represent temperatures well below and above the ferrimagnetic *T*_M_, respectively.

**Figure 2:**
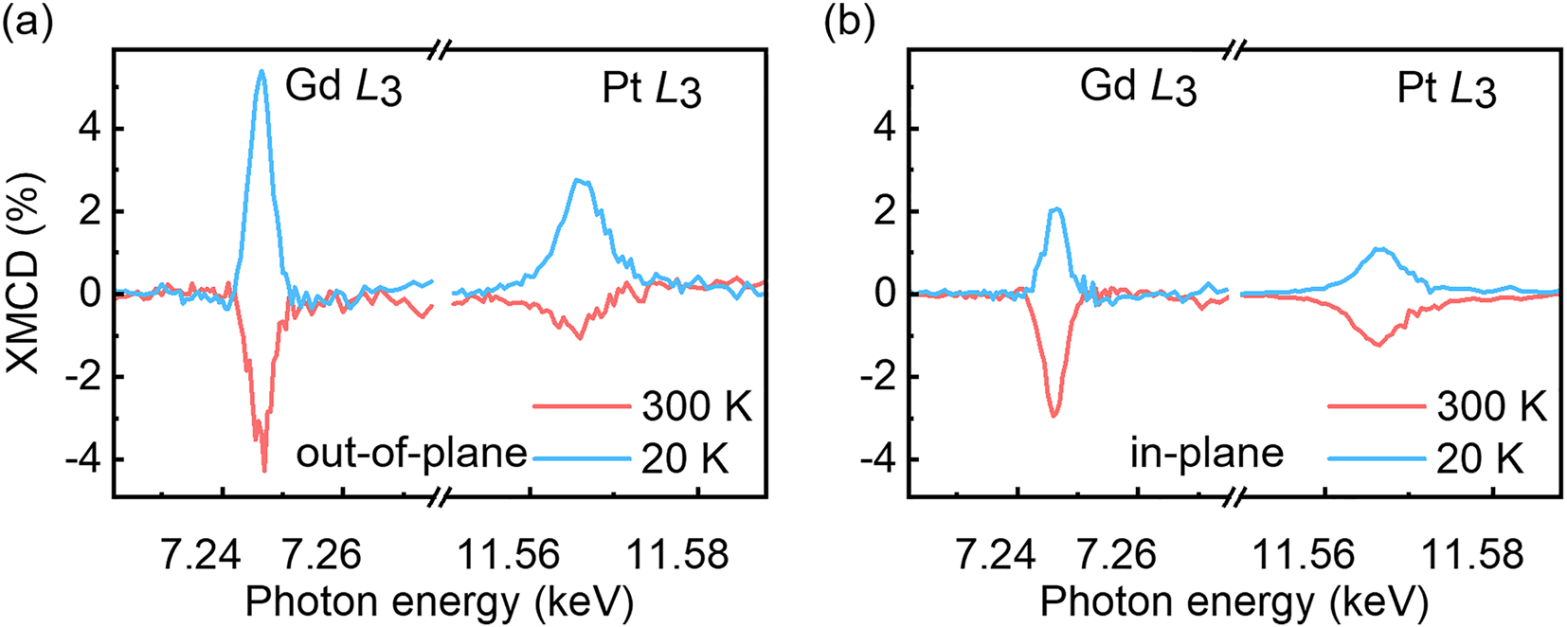
X-ray magnetic circular dichroism (XMCD) spectra of the GdFeCo/Pt film. XMCD are measured at the Gd *L*_3_- and Pt *L*_3_-edges in the presence of 0.2 T magnetic fields applied (**a**) out-of-plane and (**b**) in-plane. Measurements performed at 20 K (blue lines) and 300 K (red lines).

Figure 2 shows the XMCD measured at the Gd *L*_3_-edge (7.24 keV) and the Pt *L*_3_-edge (11.56 keV), representing the transition from the *p*-to-*d* state of each element. The out-of-plane Gd *L*_3_-edge XMCD spectra show a larger peak intensity at 20 K than at 300 K (left panel in Fig. 2a), consistent with the expected decrease of the Gd magnetic moment with temperature. While the GdFeCo films exhibit out-of-plane magnetic easy axes at all temperatures, the Gd and FeCo moments can acquire in-plane components, and hence in-plane XMCD can appear, when a 0.2 T in-plane magnetic field is applied (see Fig. 1c,d). However, in contrast to the out-of-plane XMCD, the in-plane Gd XMCD spectra show a larger peak at 300 K (left panel in Fig. 2b), implying that the in-plane component of the Gd magnetic moment is larger at 300 K. This result stems from the fact that the in-plane (hard axis) saturation field is larger at 20 K than at 300 K (see black curves in Fig. 1c,d); while the applied 0.2 T in-plane magnetic field is insufficient, at both temperatures, to completely saturate the GdFeCo magnetization along the in-plane direction, the magnetization is tilted more in-plane at 300 K. We also observe that both the out-of-plane and in-plane Gd XMCD spectra show sign reversals between 20 and 300 K. Previous studies show that when the Gd magnetic moments are aligned along the applied field, a *negative* dichroic spectrum appears at the Gd *M*_5_-edge and a *positive* dichroic spectrum appears at the *L*_3_-edge; this has been observed in ferromagnetic Gd-based materials^27–29^ and ferrimagnetic Gd-based alloys^24,30^. Thus, the positive (negative) *L*_3_-edge XMCD at 20 K (300 K) indicates that the Gd moments align parallel (anti-parallel) to the magnetic field at 20 K (300 K). See Supplementary Note 1 for the Fe XMCD spectra, which confirm the ferrimagnetic nature of the GdFeCo, i.e., the Fe and Gd moments being aligned anti-parallel. Thus, the Gd moments align parallel to the magnetic field at 20 K (below *T*_M_) and the Fe moments align parallel to the magnetic field at 300 K (above *T*_M_). The sign-flip of both the Gd and Fe XMCD across *T*_M_ is a consequence of the dominant magnetic element being interchanged at *T*_M_.

The presence of induced Pt magnetic moments in GdFeCo/Pt is evidenced by the non-zero XMCD spectra at the Pt *L*_3_-edge (right panels in Fig. 2a,b). Pt is a non-magnetic HM close enough to the Stoner criterion such that it readily acquires a magnetic moment when in contact with ferromagnetic materials^14,15,18,31^. Importantly, we observe that the Pt and Gd XMCD intensities show identical signs, at both temperatures and magnetic field directions. However, in contrast to Gd in which positive *L*_3_-edge XMCD signal indicates the moment being aligned anti-parallel to the field, a *negative* XMCD signal at the Pt *L*_3_-edge indicates the Pt moment being aligned along the applied field^19,32–34^. This suggests that the induced Pt magnetic moments in GdFeCo/Pt are always coupled anti-parallel to the Gd magnetic moments, and hence, always parallel to the FeCo magnetic moments. In other words, the induced Pt moments follow the direction of the FeCo moments, not only when the FeCo magnetic moment is larger than the Gd magnetic moment (above *T*_M_), but also when it is smaller (below *T*_M_). This alignment is observed for both out-of-plane and in-plane XMCD data (Fig. 2a,b); note that the out-of-plane (easy axis) XMCD, in particular, is obtained with a 0.2 T magnetic field which is sufficient enough for collinear out-of-plane alignment of GdFeCo which induces the Pt moment.

In fact, the parallel (anti-parallel) alignment between the induced Pt moments and the FeCo (Gd) moments were experimentally observed in Gd_100−*x−y*_Fe_*x*_Co_*y*_/Pt thin films with multiple compositions^24^. While the experimental observations of the parallel alignment of the induced-Pt and FeCo magnetic moments might seem natural following the standard interpretation given the *d–d* interactions between Pt and FeCo, what remains unclear is if the less-investigated Gd 4*f* and 5*d* magnetic moments play an active role in the MPE in this multi-elemental system. The following theoretical and experimental analysis will answer this open question. In particular, it will be shown that subtle details in the temperature-dependent magnetic depth profile, determined by XRMR, can only be explained by a model that includes the contribution from the Gd magnetic moments.

### Theoretical band repulsion model

In order to gain physical insight into the MPE in this RE–TM/HM system, we develop a simple theoretical model. Since the proximity-induced magnetic moment is a result of the hybridization of electronic states in the intermixing region (see the following section for the experimental observation of intermixing at the GdFeCo–Pt interface), we consider two electronic states, namely *A* and *B*, hybridizing with each other, where *A* and *B* may be assigned as electronic states in the Pt and those in the FeCo (or Gd), respectively. The Hamiltonian is then written as

1 $$H = \varepsilon_{A} c_{A}^{\dag } c_{A} + \varepsilon_{B} c_{B}^{\dag } c_{B} + tc_{A}^{\dag } c_{B} + t^{*} c_{B}^{\dag } c_{A} ,$$

where ${\varepsilon }_{A}$ and ${\varepsilon }_{B}$ are the energy level of the *A* and *B* states, respectively, and *t* refers to the hybridization between the two states. Straightforward diagonalization of the 2 × 2 Hamiltonian gives the perturbed energy eigenvalues, ${\widetilde{\varepsilon }}_{A}$ and ${\widetilde{\varepsilon }}_{B}$. Assuming that ${\varepsilon }_{A}>{\varepsilon }_{B}$ and keeping the lowest order terms in *t*, we obtain

2 $${\widetilde{\varepsilon }}_{A}={\varepsilon }_{A}+\frac{{\left|t\right|}^{2}}{{\varepsilon }_{A}-{\varepsilon }_{B}}, {\widetilde{\varepsilon }}_{B}={\varepsilon }_{B}-\frac{{\left|t\right|}^{2}}{{\varepsilon }_{A}-{\varepsilon }_{B}}.$$

We find two important observations in this equation. First, ${\widetilde{\varepsilon }}_{B}<{\varepsilon }_{B}<{\varepsilon }_{A}<{\widetilde{\varepsilon }}_{A}$ is satisfied regardless of the microscopic mechanism of the hybridization *t*. Therefore, one can conclude that, in most situations, the energy difference between the two states increases when the hybridization is turned on. We call this the hybridization-driven *band repulsion*. Second, since the strength of the band repulsion $\propto {\left|t\right|}^{2}/({\varepsilon }_{A}-{\varepsilon }_{B})$, the repulsion becomes more significant when the energy difference $\left({\varepsilon }_{A}-{\varepsilon }_{B}\right)$ is small. These two remarks hold even when *t* goes beyond the perturbative regime.

**Figure 3:**
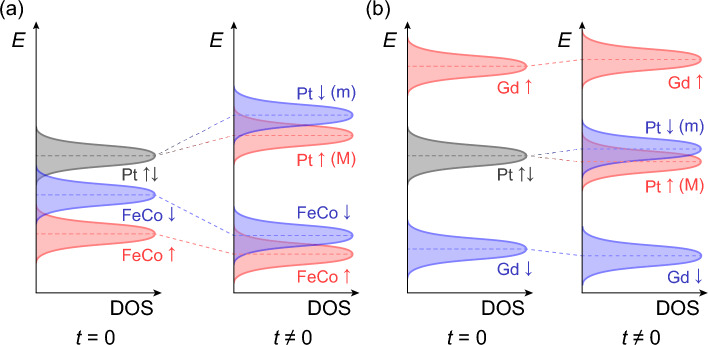
Mechanism of magnetic proximity effect based on the band repulsion model. The magnetic moments in Pt are induced by (**a**) FeCo and (**b**) Gd. Each panel illustrates how the energies (*E*) of the electronic states evolve when the hybridization (*t*) is turned on. M and m refer to the majority and minority bands of the spin-split Pt bands, respectively. The principle of band repulsion implies that the FeCo and Gd elements induce proximity-induced magnetism in the same direction, despite the antiferromagnetic alignment of the FeCo and Gd moments. All bands are drawn with the same heights of the density of states (DOS) for simplicity, and thus their magnitudes have no meaning here.

Now we apply the principle of band repulsion to the MPE. In the regime of perturbative *t*, one may separately calculate contributions from the Gd and FeCo and add them up. For FeCo, the main contributions come from *d* states whose majority (↑) and minority (↓) bands are partially occupied (Fig. 3a). Since the majority band is located below the minority band, the spin-↑ Pt state suffers a stronger band repulsion than the spin-↓ Pt state, and thus the energy level of the electrons in the Pt becomes spin-split. Here, the energy is larger for spin-↓ such that the induced magnetic moment prefers the same direction as the magnetic moment of FeCo.

Next, we consider the Gd case. For Gd, the magnetic moment mainly originates from *f* states whose majority (↓) band is far below the Fermi level and minority (↑) band is above the Fermi level^35^ (here, we assign the spin directions opposite to the FeCo case given the antiferromagnetic configuration). For this situation, the principle of the band repulsion implies that the energy of the spin-↑ Pt state shifts downward while the spin-↓ Pt state shifts upward (Fig. 3b). Remarkably, Gd elements induce magnetic moments in the Pt which are *opposite* to the direction of their own magnetic moments. In other words, the effects of Gd and FeCo are additive although their own magnetic moments are antiferromagnetically coupled. It should be noted that real situations can be more complex than our simplified illustration, as they may involve various factors such as multiple contributing bands and band- or spin-dependent hopping. In addition, the Gd 5*d* states, which are spin-polarized by the Gd 4*f* states and mediates the antiferromagnetic configuration in GdFeCo, may also contribute to the induced Pt magnetic moments being anti-parallel to the Gd magnetic moments. Materials that exhibit behaviors beyond the scope of our model would necessitate a more comprehensive investigation into the specific circumstances.

While our model suggests that the effects of the FeCo and Gd are accumulative, it remains to be experimentally proven that the electronic states of both FeCo and Gd indeed affect the Pt electronic states; our XMCD results can still be understood even if only the FeCo plays an active role in the inducing the Pt magnetic moment. In order to verify the effects of both the RE and TM in this system, we take advantage of the fact that our model predicts different penetration depths for each element. The induced spin-split states near the interface would penetrate into the Pt bulk within a certain length scale, which is roughly proportional to the characteristic coherence length *v*_F_ × ℏ*/ΔE*, where *v*_F_ is the Fermi velocity and *ΔE* is the amount of the energy shift between the spin-split states as depicted in Fig. 3. Hence, we expect that the magnetic moment induced by Gd atoms (for which *ΔE* is smaller) would penetrate deeper into the Pt bulk. Exploiting the distinct temperature-dependence of the Gd and FeCo magnetic moments, we can infer that the induced Pt moment will persist further from the GdFeCo-Pt interface below *T*_M_, where the Gd moment is larger than FeCo moment, than above. Therefore, experimental observation of temperature-dependent change in the MPE penetration depths would support the effects of both the RE and TM, and particularly the prominent role of the Gd 4*f* electronic states.

### Depth profile of the induced Pt magnetic moment

**Figure 4:**
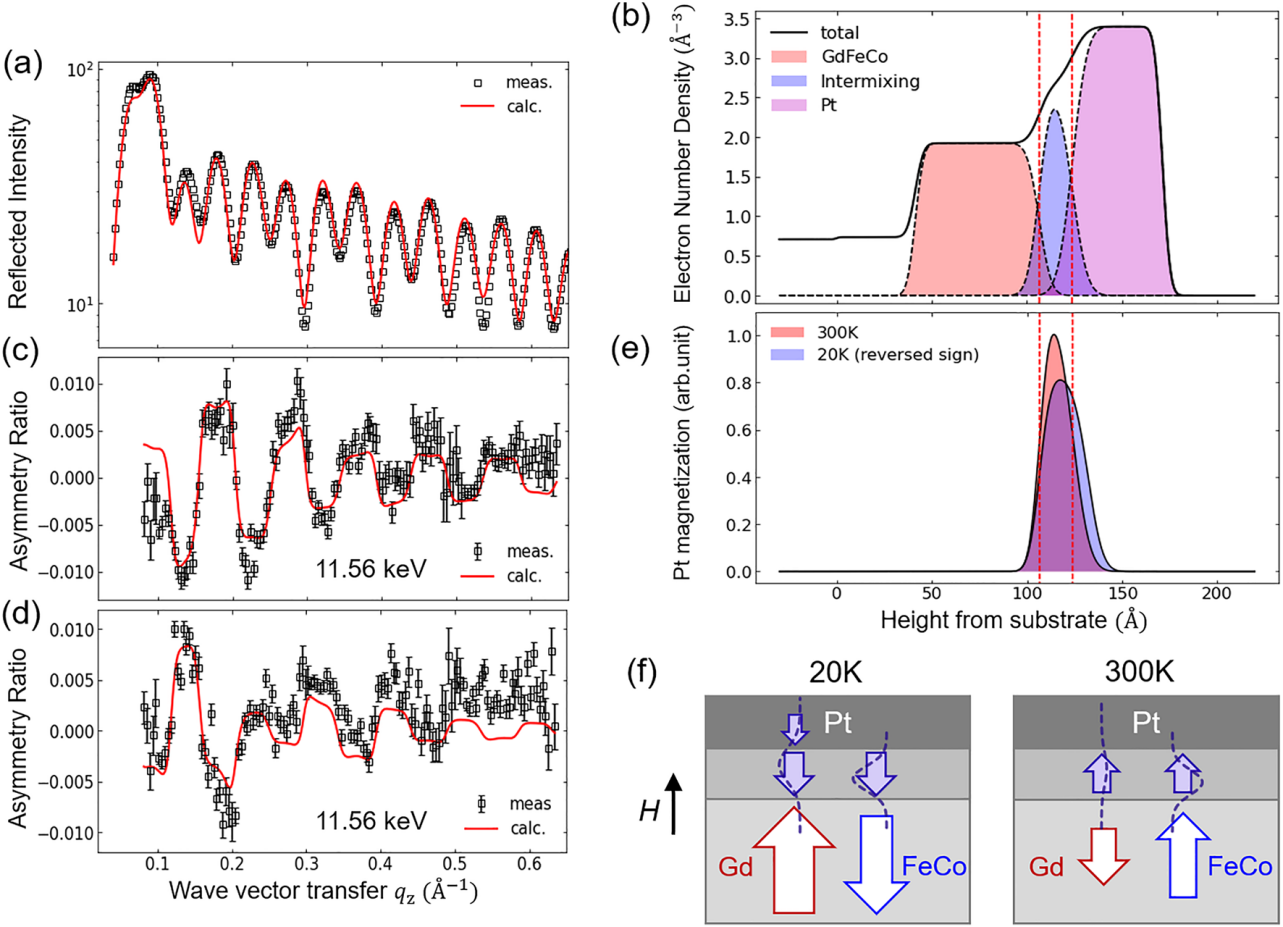
Depth profile of the induced Pt magnetic moments. (**a**) XRR data and curve-fitting results. The wave vector transfer ${q}_{z}$ is the difference between wave vectors of incident and scattered x-rays. Black empty squares (□) are experimental data, and the red solid line represents the best-fit. (**b**) Electron number density plotted using the structural parameters obtained from the best-fit results. Black solid line represents the total electron density, and the electron densities of each layer are shown by shaded areas of different colors. (**c**, **d**) XRMR AR data measured at (**c**) 300 K and (**d**) 20 K and curve-fitting results. The XRMR is measured at the Pt *L*_3_-edge (11.56 keV). (**e**) Magnetic depth profiles plotted using the best-fit parameters from (**c**) and (**d**). The magnetic profile corresponding to 20 K is plotted with reversed sign. The vertical red dotted lines in (**b**) and (**e**) indicate the locations of GdFeCo/intermixing and intermixing/Pt interfaces. (**f**) Summary of XRR and XRMR analysis. The Pt magnetic moment is indicated by purple arrows. The Gaussian-shaped dotted curves depict the penetration of the Gd-induced and FeCo-induced spin-split states. At 300 K, the induced Pt moment is present only in the GdFeCo–Pt intermixing layer, and at 20 K, the induced Pt moment persists into the pure Pt region. Note that 20 K < *T*_M_ < 300 K, such that the Gd (FeCo) moments are aligned with the magnetic field at 20 K (300 K).

In order to confirm the effect of Gd and uncover the additive roles of the proximity-induced magnetic moments with two different origins, we investigate the interfacial structural and magnetic properties of the Pt layer using x-ray reflectivity (XRR) and XRMR. First, XRR is used to determine the structural depth profile^36–38^. XRR data are quantitatively analyzed by fitting it with multiple parameters such as the film thickness, density, and interfacial roughness (Fig. 4a). From the XRR analysis (see Supplementary Note 2), we find the presence of significant intermixing at the GdFeCo–Pt interface; the thickness of the intermixing layer is found to be ~ 17.5 Å. Figure 4b shows the depth profile of the electron number density obtained using the best-fit parameters.

The XRMR intensities of GdFeCo/Pt are measured at the *L*_3_ absorption edge of Pt, which are used to determine the depth profile of the Pt magnetic moment induced by the ferrimagnetic GdFeCo magnetic layer. Previous studies show that XRMR can be successfully utilized for the determination of the element-specific magnetic depth profile^24,39–45^. While the GdFeCo/Pt shows strong PMA, XRMR is measured with a 0.2 T in-plane magnetic field, since the XRMR magnetic scattering factor is much more sensitive to in-plane magnetization than the out-of-plane magnetization (see Methods). The depth profile of the induced Pt magnetic moments is obtained by fitting the XRMR asymmetry ratio (AR) curves (Fig. 4c,d). See Supplementary Note 3 for details on XRMR AR fitting. Figure 4e shows the depth profiles of the induced Pt magnetic moments at 300 K and 20 K, obtained using the best-fit parameters of the induced magnetic layers. Note that the Pt magnetic profile at 20 K (purple shaded area in Fig. 4e) is plotted with reversed sign for better visualization. That is, the directions of the induced Pt magnetic moments at temperatures above (300 K) and below (20 K) the GdFeCo ferrimagnetic *T*_M_ are opposite to each other, in agreement with the XMCD results in Fig. 2. We note that the direct quantitative comparison of the Pt magnetization (area ratio or peak height) between the two temperatures is complicated, as discussed with the in-plane XMCD results (Fig. 2b). The GdFeCo magnetization is tilted more in-plane at 300 K than at 20 K with an applied 0.2 T in-plane field, which results in the induced Pt moment being seemingly larger at 300 K.

Setting aside the peak height comparison, we find a notable difference between the shapes of the depth profiles of the Pt magnetization at 300 K and 20 K (Fig. 4e): the depth-resolved Pt magnetization shows a wider profile at 20 K. Given the existence of the structural intermixing layer, the induced Pt moment can originate from two distinct regions within the Pt layer, (1) the structural intermixing region, and (2) the pure Pt region adjacent to the intermixing region. The magnetic depth profile at 300 K (pink shaded region in Fig. 4e) shows that the induced Pt magnetic moment mostly originates from the intermixing region with the induced moment in the pure Pt region being negligible. On the other hand, at 20 K, the magnetic depth profile at 20 K (purple shaded region in Fig. 4e) shows appreciable Pt magnetization in the pure Pt region, with the width of this induced magnetic layer being ≈ 8 Å (see Supplementary Note 3). The difference in the induced Pt magnetic moment depth profiles at the two representative temperatures below and above *T*_M_ is schematically depicted in Fig. 4f. We note that it is highly possible that the Pt magnetization observed in the GdFeCo–Pt intermixing region originates from a Pt-embedded-GdFeCo magnetic alloy rather than a purely proximity-induced magnetism of a non-magnetic material. We suggest follow-up studies on Gd XRMR magnetic depth profile analysis, which might shed light on the exact origin of the Pt magnetism in the GdFeCo–Pt intermixing region. Nevertheless, the existence of the Pt magnetic moments in the pure Pt region at 20 K can be considered to be the MPE in the conventional sense.

If the Pt magnetic moments are solely induced by the FeCo magnetic moments, it is difficult to conceive the induced Pt moments having different depth profiles at temperatures below and above *T*_M_. We suspect that this intriguing behavior originates from the effect of Gd. As discussed earlier, the Gd moments possess longer MPE penetration depths compared to the FeCo moments. At 20 K, the magnetic moment of Gd becomes larger, such that the proximity-induced magnetic states can penetrate deeper into the Pt bulk. That is, the different Pt magnetic depth profiles at the two temperatures are a combined effect of the Gd and FeCo magnetic moments having distinct temperature-dependence, and the Gd-induced and FeCo-induced Pt spin-split states having different penetration lengths. Furthermore, the penetration depth being longer than the intermixing range at 20 K also supports our theoretical model, taking into account the mediation by the conduction electrons in Pt. Therefore, the XRMR measurements and analysis confirm that the Gd electronic states indeed play a prominent role in the proximity-induced magnetism in Pt and support our picture for additive contributions of Gd and FeCo to the MPE in the GdFeCo/Pt system.

## Conclusions

Our experimental results and theoretical model reveal that in an archetype RE–TM/HM system, the GdFeCo/Pt bilayer, the Gd and FeCo work in-phase to make the proximity-induced Pt magnetic moment align with the FeCo moment, regardless of net GdFeCo moment direction. Our theoretical model reveals that the induced moment direction is determined by the relative order of the energy levels of Gd and FeCo. The apparent decoupling between the induced Pt moment and the net GdFeCo moment highlights the additive roles of the antiferromagnetically-exchange-coupled RE and TM elements and also suggests induced HM magnetic moments can be non-zero even when the ferrimagnetic moment is compensated, opening up the possibility of unconventional antiferromagnetic spintronics phenomena near the ferrimagnetic compensation. Our findings demonstrate the nature of the elemental magnetic coupling in RE–TM/HM systems and invite further studies to investigate the applicability of the current conclusions to other RE–TM/HM systems.

## Methods

### Thin film growth

Thin film samples are grown by DC magnetron sputter deposition. The base pressure of the deposition chamber is 1 × 10^−8^ Torr. The film structure consists of SiN(5 nm)/Gd_23_Fe_67.4_Co_9.6_(8 nm)/Pt(5 nm) deposited on a Si substrate with a thermally oxidized SiO_2_(100 nm) layer.

### X-ray measurements

The hard x-ray (Gd *L*-edge and Pt *L*-edge) measurements shown in Figs. 2 and 4 are conducted at the Advanced Photon Source 4-ID-D beamline at Argonne National Laboratory. At the 4-ID-D beamline, a diamond phase plate is used to make the helicity of the incident x-rays parallel or anti-parallel to the applied magnetic field, enabling XMCD and XRMR measurements. The energy of the incident x-rays is tuned to the Gd and Pt *L*_3_ absorption edges at 7.24 keV and 11.56 keV, respectively, and the measurement temperatures are 300 K and 20 K. The XMCD measurements are performed using the fluorescence detection mode, and fixed (0.2 T) out-of-plane and in-plane magnetic fields are applied. For XRMR, the reflected intensities (${I}_{+}$ and ${I}_{-}$) are measured with the helicities of the circularly polarized x-ray parallel (${\mu }^{+}$) and antiparallel (${\mu }^{-}$), respectively, to the magnetic field. The asymmetry ratio AR ≡ $({I}_{+}-{I}_{-})/({I}_{+}+{I}_{-})$ obtained from the XRMR intensities are shown in Fig. 4c,d. The XRMR experiments are performed with in-plane magnetic field for the following reason. At the Pt *L*_3_ edge energies (hard x-ray region), XRMR is measured at grazing angles, such that the magnetic scattering factor of XRMR, $({\widehat{\upepsilon }}_{f}\times {\widehat{\epsilon }}_{i})\cdot \widehat{M}$, is much more sensitive to the magnetization in the in-plane direction than in the out-of-plane direction (${\widehat{\epsilon }}_{i}, {\widehat{\epsilon }}_{f}$ are the polarization vectors of the incident and scattered x-rays, respectively, and $\widehat{M}$ is the magnetization vector of the sample). The XRR and XRMR analysis methods are explained in Supplementary Notes 2 and 3.

The soft x-ray (Gd *M*-edge and Fe *L*-edge) XMCD measurements shown in Supplementary Note 1 are conducted at the Pohang Light Source 6A beamline. The energies of the incident x-rays are tuned to the Gd *M*_4,5_ and Fe *L*_2,3_ absorption edges, and the measurement temperatures are 300 K and 10 K. The measurements are done in total electron yield mode.

### Supplementary Information

Supplementary Information.

## Data Availability

The data in the current study are available from the corresponding authors on request.
