# Supplementary material for: Additive roles of antiferromagnetically coupled elements in the magnetic proximity effect in the GdFeCo/Pt system
Source: Sci Rep. 2024 Apr 25;14:9476. doi: 10.1038/s41598-024-60076-9 (PMC11043343; doi:10.1038/s41598-024-60076-9)
Supplement: Supplementary file 1 — Supplementary Information. [file 41598_2024_60076_MOESM1_ESM.docx]

Supplementary Information

Additive roles of antiferromagnetically coupled elements in the magnetic proximity effect in the GdFeCo/Pt system

Jung Yun Kee ^1,2,†^, Kook Tae Kim ^2,†^, In Hak Lee ^1,†^, Ilwan Seo ^2^, Jun-Young Chang ^1,3^, Ah-Yeon Lee ^4^, Woo-suk Noh ^5^, Young Jun Chang ^6^, Seung-Young Park ^7^, Sug-Bong Choe ^3^, Duck-Ho Kim ^1^, Kyoung-Whan Kim ^1,8^*, Yongseong Choi ^9,^*, Dong Ryeol Lee ^2,^*, Jun Woo Choi ^1,^*

*^1^ Center for Spintronics, Korea Institute of Science and Technology (KIST), Seoul 02792, Korea*

*^2^ Department of Physics, Soongsil University, Seoul 06978, Korea*

*^3^ Department of Physics and Astronomy, Seoul National University, Seoul 08826, Korea*

*^4^ Center for Research Equipment, Division of Scientific Instrumentation & Management, Korea Basic Science Institute (KBSI), Daejeon 34133, Korea*

^5^ Korea Foundation for Max Planck POSTECH/Korea Research Initiative, Pohang, 37673, Korea

^6^ Department of Physics, University of Seoul, Seoul 02504, Korea

*^7^ Center for Scientific Instrumentation, Division of Scientific Instrumentation & Management, Korea Basic Science Institute (KBSI), Daejeon 34133, Korea*

*^8^ Department of Physics, Yonsei University, Seoul 03722, Korea*

*^9^ Advanced Photon Source, Argonne National Laboratory, Argonne, Illinois 60439, USA*

^†^ These authors equally contributed to this work.

Jung Yun Kee, Kook Tae Kim, In Hak Lee

* Corresponding authors.

kwkim@yonsei.ac.kr, ychoi@anl.gov, drlee@ssu.ac.kr, junwoo@kist.re.kr

**Supplementary Note 1****. Antiferromagnetic coupling between the Gd and Fe magnetic moments in GdFeCo**

In order to confirm the ferrimagnetism in GdFeCo, we measure x-ray magnetic circular dichroism (XMCD) spectroscopy at the Gd *M*-edge and Fe *L*-edge. The measurements are performed at 300 K and 10 K, respectively, which represent temperatures above and below the ferrimagnetic compensation temperature *T*_M_, respectively. In Fig. S1, we see that the Gd and Fe XMCD signals have opposite signs for both temperatures, implying that the Gd and Fe magnetic moments are always aligned anti-parallel. This again confirms that the GdFeCo is ferrimagnetic. The negative (positive) XMCD signal at the Fe *L*_3_-edge at 300 K (10 K) indicate that the Fe aligns parallel (anti-parallel) to the magnetic field. While we do not measure the XMCD at the Co *L*-edge in this study, we refer to an earlier study which explicitly confirms that the XMCD signals show identical signs at the Co and Fe *L*-edges in GdFeCo^1^, i.e., the Fe and Co moments are parallel-aligned in GdFeCo.


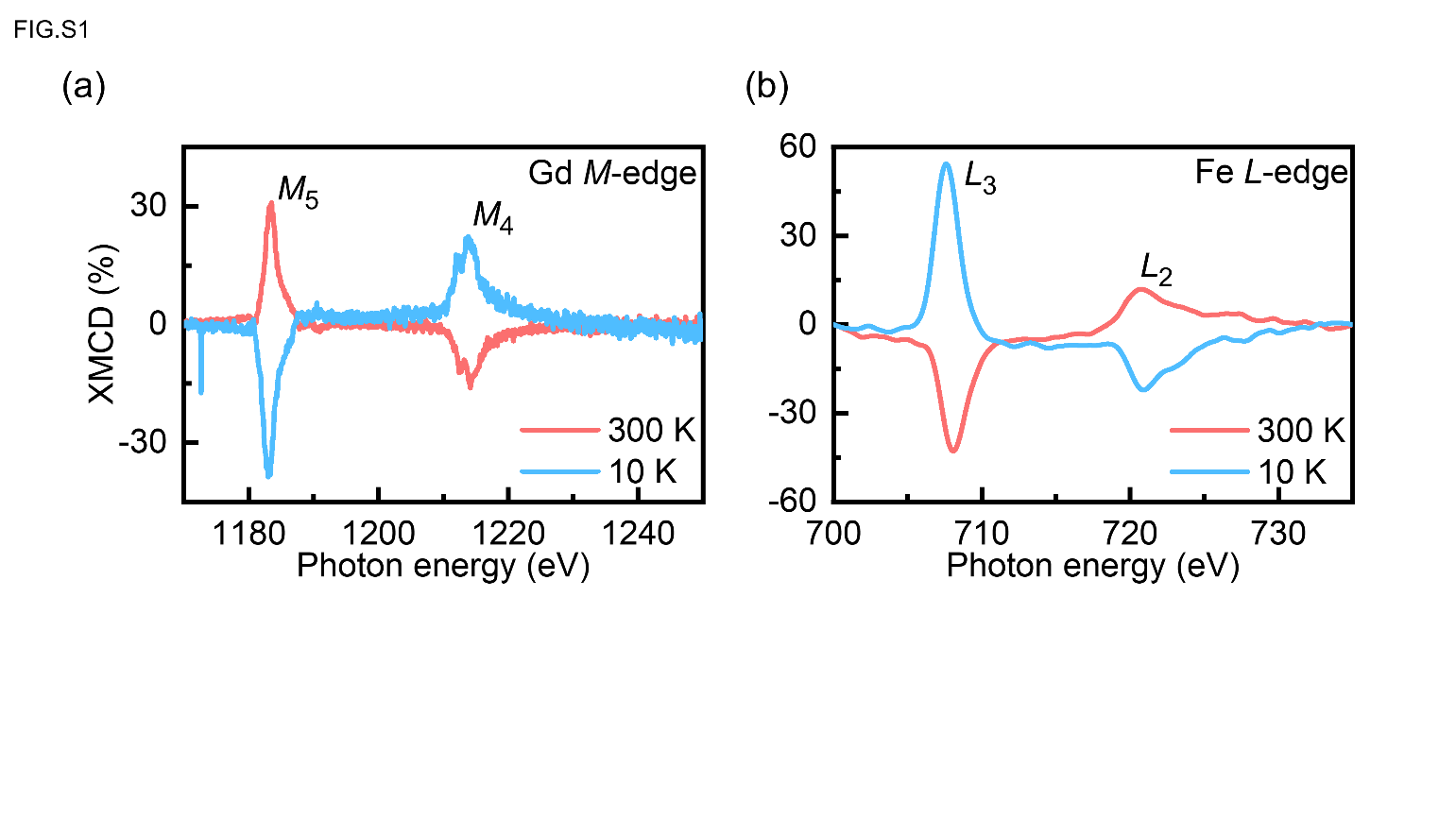


**Figure S1.** **X-ray magnetic circular dichroism (XMCD) spectra of the GdFeCo/Pt film.** XMCD measured at (**a**) the Gd *M*-edge and (**b**) the Fe *L*-edge.

**Supplementary Note 2. X-ray reflectivity (XRR) data analysis**

As the first step to analyze the XRR data shown in main text Fig. 4a, we use a well-known dynamical calculation method, the distorted wave Born approximation based on dynamical calculation, to calculate the expected XRR with certain material parameters^2,3^. This is a method that considers the chemical or magnetic height fluctuations at the interface (i.e., interface roughness) through perturbation using eigenstates that solve Maxwell's equation for the susceptibility χ of each layer from the model parameters representing the sample and the atomic form factors of the elements comprising each layer.

We then fit the calculated XRR to the experimental data to find the best-fit material parameters. In order to check whether the difference between the curve fitting of model calculations and experimental values is a global minimum, methods that calculate the probability distribution of fitting parameters using neural networks or Bayesian inference methods have been mainly used in recent years^2,4,5^. Here, the latter method is used. The Bayesian curve fitting is a method to obtain the probability distribution $P(\text{parameter}|\text{data})$ (called posterior) of each parameter based on the measured data^2,6^. For Bayesian inference, we used the Markov chain Monte Carlo (MCMC) method. The MCMC method allows us to obtain samples of a parameter, known as a Markov Chain, which contains all the information about the posterior distribution of the parameter. For this purpose, the python *lmfit* package is used^7^. In our study, we performed 3,000,000 samplings to obtain the Markov chain and then selected only one sample per three to avoid similarity between the samples. Also, the first 20,000 samples are excluded to obtain a stable Markov chain.

Using this Bayesian inference method, it is possible to obtain globally optimized parameters that best describe data within the range of parameters given in advance, credible intervals of each parameter, and cross correlation between parameters at once^2,6^. upon performing the curve fitting of the XRR data using Bayesian inference, one can obtain the probability distribution of the free parameters of the structural model. The posterior distribution of the parameters and a credible interval for the best-fit parameters are shown in Fig. S2 and Table S1, respectively. These parameters are used for calculated best-fit curve shown in main text Fig. 4a (solid red curve).

In Fig. S2 and Table S1, we see the existence of an GdFeCo-Pt interfacial intermixing layer. The dotted red line in Fig. S3 shows the calculated best-fit of the XRR data assuming a structural model with an ideal, well-defined GdFeCo-Pt interface, i.e., no interfacial intermixing. Despite the fact that the Bayesian inference method finds a globally optimized best-fit parameter, there is a clear difference between the calculated best-fit curve and the experimental data, indicating that the structural model with no interfacial intermixing cannot fully explain the XRR data. Alternatively, we consider a structural model with an intermixing layer at the interface between the GdFeCo and Pt; such intermixing typically occurs at the interface of sputter-deposited metallic films. The thickness of the GdFeCo-Pt intermixing layer is considered a free fit parameter, but the refractive index of this layer is fixed to the average value of the GdFeCo and Pt layers. The fitting results using the new structural model (solid black curve in Fig. S3) show that it explains the XRR data much better than the model with no intermixing.

**
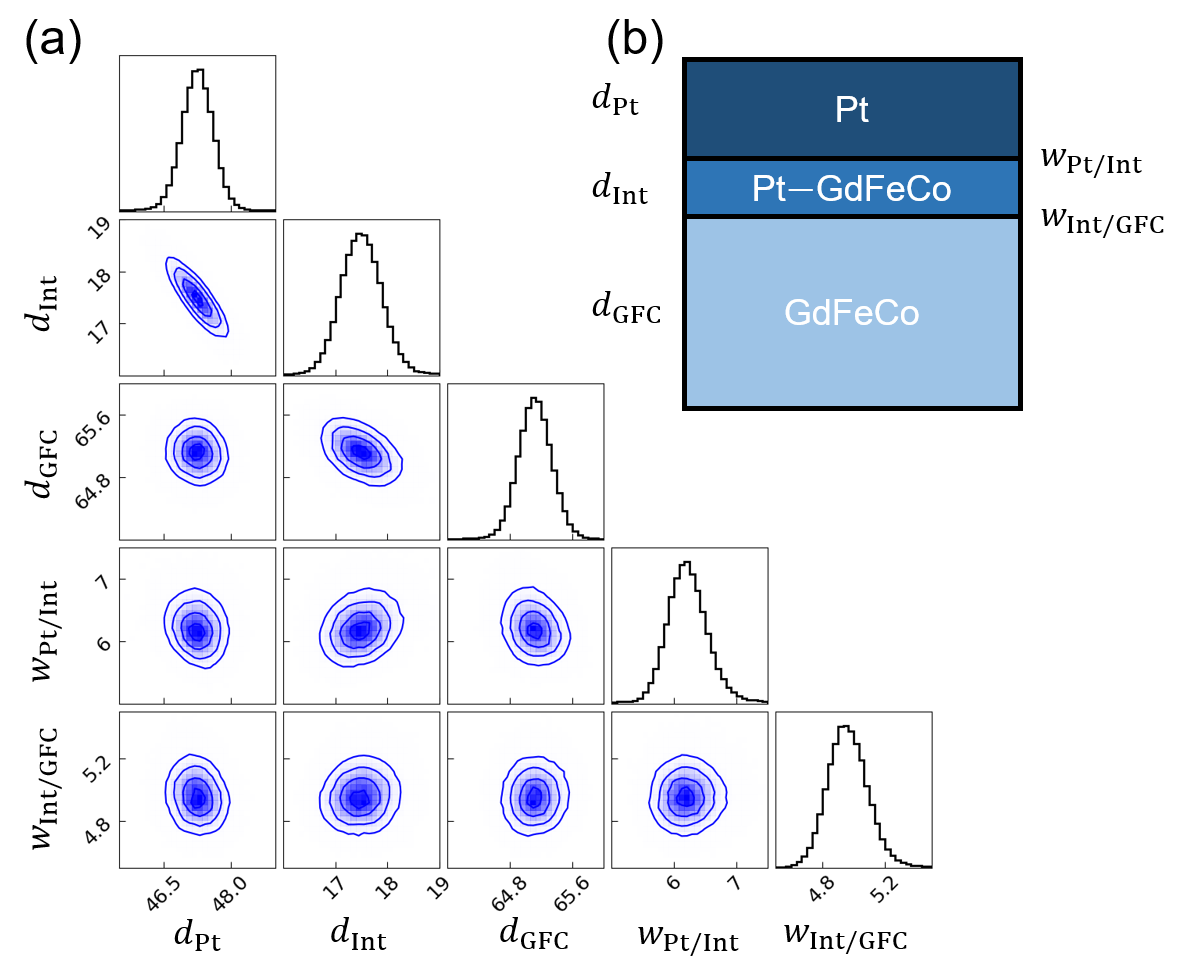
**

**Figure S2. X-ray reflectivity (XRR) analysis.** (**a**) Posterior distributions of parameters obtained from the XRR curve fitting of main text Fig. 4a. *d* and *w* are the layer thickness and interfacial roughness, respectively. The black histogram shows the posterior distribution of each parameter alone, and the blue contour plot shows the correlation between the parameters. (**b**) Schematic diagram of the structural model of the sample used in the XRR fitting. The intermixing layer between the Pt and GdFeCo layers is labeled as Pt-GdFeCo.

| **Parameter** | $\boldsymbol{-1\sigma(Å)}$ | **Median** | $\boldsymbol{+1\sigma}$ $\mathbf{(Å)}$ |
| --- | --- | --- | --- |
| $r_{\mathrm{Pt}}$ | -0.0044 | 0.8277 (a.u) | 0.0040 |
| $r_{\mathrm{GdFeCo}}$ | -0.0314 | 0.9215 (a.u) | 0.0245 |
| $d_{\mathrm{Pt}}$ | -0.4072 | 47.2554$(Å)$ | 0.04027 |
| $d_{\mathrm{int}}$ | -0.4521 | 17.4759$(Å)$ | 0.4521 |
| $d_{\mathrm{GdFeCo}}$ | -0.2619 | 65.111$(Å)$ | 0.2494 |
| $w_{air/Pt}$ | -0.2179 | 3.2555$(Å)$ | 0.6012 |
| $w_{Pt/int}$ | -0.0523 | 6.1794$(Å)$ | 0.0632 |
| $W_{int/GFC}$ | -0.1328 | 4.9581$(Å)$ | 0.1794 |

**Table S1.** **Best-fit parameters of the x-ray reflectivity (XRR) data.** *r, d,* and *w* denote relative density, thickness, and interfacial roughness, respectively. These parameters are used for calculated best-fit curve shown in main text Fig. 4a and Fig. S3 (black curves).

**
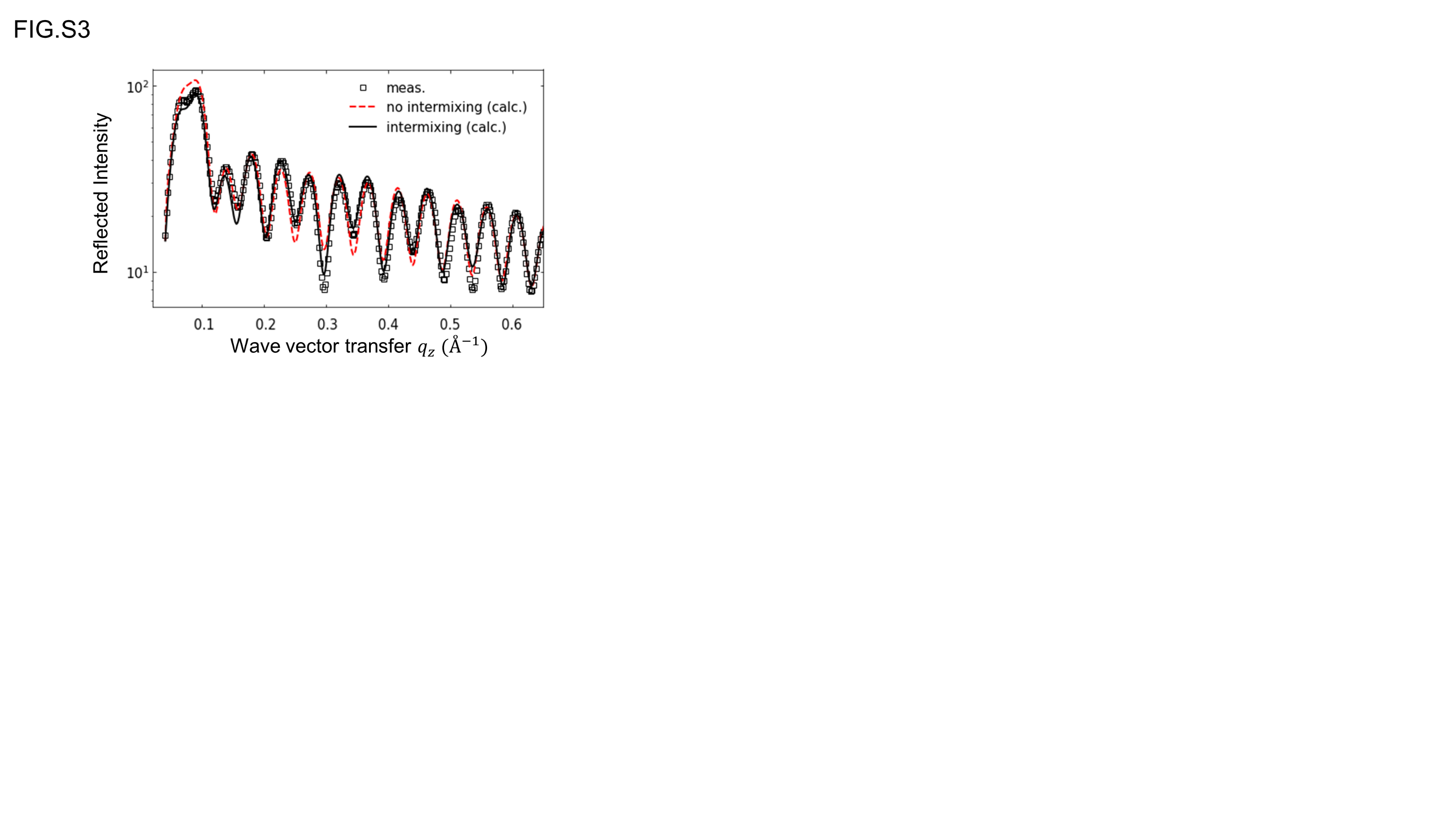
**

**Figure S3.** **X-ray reflectivity (XRR) data and curve-fitting results.** Black empty squares (**□**) are experimental data, and the dotted red and black solid lines represent the best fits without and with the intermixing layer, respectively.

**Supplementary Note 3. X-ray resonant magnetic reflectivity (XRMR) data analysis**

The XRMR asymmetry ratio (AR) data are curve-fitted to obtain the quantitative depth profile of the induced Pt magnetic moments. Similar to the XRR data analysis, the model calculation of the AR is performed using the dynamical XRMR calculation method^3,6^ (see Supplementary Note 2). Since the structure of the sample is already known from the XRR analysis, the free parameters for the curve fitting of the AR are only related to the Pt magnetic properties. Given the existence of the GdFeCo–Pt intermixing layer, the induced Pt moment can originate from two distinct regions: (1) the induced magnetic layer of Pt in the intermixing region, and (2) the induced magnetic layer in the pure Pt region near the interface with the intermixing region. The total induced Pt magnetic moment is the sum of the contributions of these two magnetic layers.

Using the electron depth profile obtained from XRR and the magnetic layer model described above, the AR data obtained at both temperatures are curve-fitted (main text Fig. 4c, d). Once again, the Bayesian inference method (see Supplementary Note 2) is used. The fitting results are shown in Fig. S4 for posterior distribution and Table S2 for best-fit parameters. For the AR fitting at 300 K, the best fit is obtained by assuming no magnetic layer in the pure Pt region, suggesting that the induced Pt magnetic moment mostly originates from the intermixing region with the magnetic moment induced in the pure Pt region being negligible. At 20 K, the best fit shows the existence of an induced magnetic layer in the pure Pt region, with the width of this magnetic layer being ≈ 8 Å (Fig. S4 and Table S2). We note that the reflectivity data can only be fitted properly assuming the presence of a “magnetic intermixing layer”. When the intermixing thickness is not included as a parameter, the calculated interference pattern positions do not change such that the best-fit of the XRMR data cannot be obtained.

**
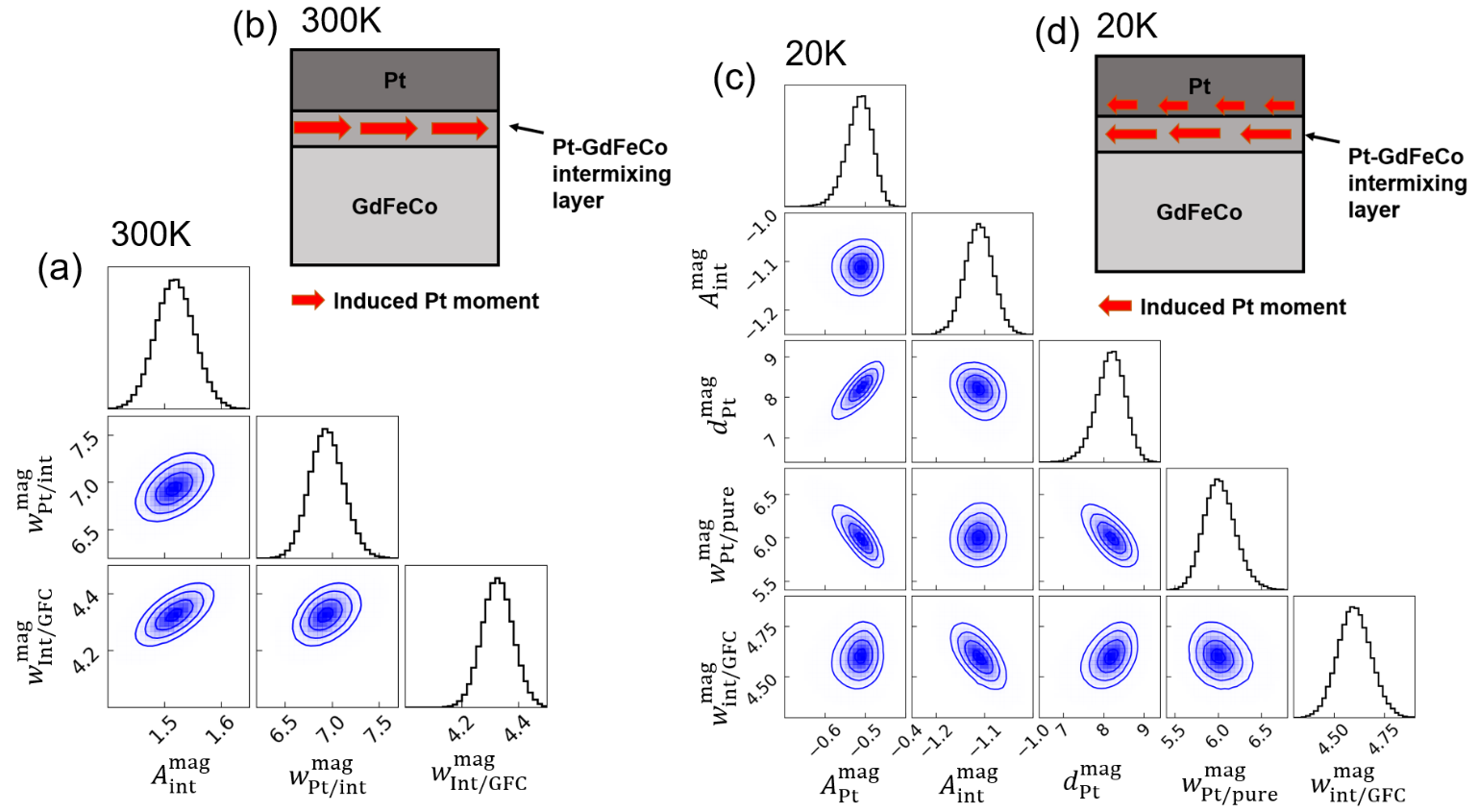
**

**Figure S4. X-ray resonant magnetic reflectivity (XRMR) analysis.** (**a**) Posterior distributions of the parameters resulting from the best-fit to the XRMR measured at 300 K. The magnetic field is applied in the in-plane direction. *A*^mag^ and *w*^mag^ denote magnetic amplitude and magnetic interfacial roughness, respectively. (**b**) Schematic representation of the structural model used in the fitting in (**a**). It can be seen that the magnetic moment is mostly induced on the Pt atoms in the intermixing layer between the GdFeCo and Pt layers. (**c**) Posterior distributions of the parameters resulting from the best-fit to the XRMR measured at 20 K. (**d**) Schematic representation of the structural model used in the fitting in (**c**). At 20 K, magnetic moments induced even in the pure Pt layer appear, and the direction of the magnetic moments is reversed compared to the 300 K case.

***T* = 300 K**

| **Parameter** | $\boldsymbol{-1\sigma(Å)}$ | **Median** | $\boldsymbol{+1\sigma(Å)}$ |
| --- | --- | --- | --- |
| $A_{\mathrm{int}}^{\mathrm{mag}}$ | -0.0341 | 1.5180 (a.u) | 0.0347 |
| $w_{Pt/int}^{\mathrm{mag}}$ | -0.1759 | 6.941$(Å)$ | 0.1844 |
| $w_{int/GFC}^{\mathrm{mag}}$ | -0.0542 | 4.325$(Å)$ | 0.0552 |

***T* = 20 K**

| **Parameter** | $\boldsymbol{-1\sigma}$ $\mathbf{(Å)}$ | **Median** | $\boldsymbol{+1\sigma(Å)}$ |
| --- | --- | --- | --- |
| $A_{\mathrm{pt}}^{\mathrm{mag}}$ | -0.0349 | -0.5143 (a.u) | 0.0297 |
| $A_{\mathrm{int}}^{\mathrm{mag}}$ | -0.0290 | -1.1117 (a,u) | 0.0285 |
| $d_{\mathrm{Pt}}^{\mathrm{mag}}$ | -0.3769 | 8.1745$(Å)$ | 0.3362 |
| $w_{Pt/pure}^{\mathrm{mag}}$ | -0.1736 | 6.0135$(Å)$ | 0.1958 |
| $w_{pure/int}^{\mathrm{mag}}$ | Fixed with the same value as $w_{Pt/int}^{\mathrm{mag}}$ of 300K | | |
| $w_{int/GFC}^{\mathrm{mag}}$ | -0.0801 | 4.600$(Å)$ | 0.0832 |

**Table S2. Best-fit parameters of the x-ray resonant magnetic reflectivity (XRMR) data.** *A*^mag^ and *w*^mag^ denote the magnetic moment amplitude and magnetic interfacial roughness of the magnetic layer, respectively. These parameters are used for calculated best-fit curve shown in main text Fig. 4c, d.

**References**

1. Woo, S. et al. Current-driven dynamics and inhibition of the skyrmion Hall effect of ferrimagnetic skyrmions in GdFeCo films. *Nat. Commun.* **9**, 959 (2018).
2. Nelson, A. R. J. & Prescott, S. W. *refnx*: neutron and X-ray reflectometry analysis in Python. *J. Appl. Crystallogr.* **52**, 193-200 (2019).
3. Lee, D. R. et al. X-ray resonant magnetic scattering from structurally and magnetically rough interfaces in multilayered systems. II. Diffuse scattering. *Phys. Rev. B* **68**, 224410 (2003).
4. Kim, K. T. & Lee, D. R. Probabilistic parameter estimation using a Gaussian mixture density network: application to X-ray reflectivity data curve fitting. *J. Appl. Crystallogr.* **54**, 1572-1579 (2021).
5. Kim, D.-O. et al. Asymmetric magnetic proximity effect in a Pd/Co/Pd trilayer system. *Sci. Rep.* **6**, 25391 (2016).
6. Kim, K. T. et al. X-ray reflectivity data analysis using Bayesian inference: The study of induced Pt magnetization in Pt/Co/Pt. *Curr. Appl. Phys.* **30**, 46-52 (2021).
7. Newville, M., Stensitzki, T., Allen, D. B. & Ingargiola, A. LMFIT: Non-Linear Least-Square Minimization and Curve-Fitting for Python (0.8.0). *Zenodo.* DOI: 10.5281/zenodo.11813 (2014).
